# The phenomenological-existential comprehension of chronic pain: going beyond the standing healthcare models

**DOI:** 10.1186/1747-5341-9-2

**Published:** 2014-01-10

**Authors:** Daniela Dantas Lima, Vera Lucia Pereira Alves, Egberto Ribeiro Turato

**Affiliations:** 1 Rua Presidente Bernardes 1293 ap.43 Jd. Flamboyant, Campinas CEP 13091-160, SP, Brasil; 2 Av. Julio de Mesquita 536 ap. 32 Cambuí, Campinas CEP 13025-061, SP, Brasil; 3 Departamento de Psicologia Médica e Psiquiatria FCM/UNICAMP, Caixa Postal 6111, CEP 13083-970 Campinas, SP, Brasil

**Keywords:** Intractable pain, Psychosomatic, Hospital out patient clinic, Psychological stress, Social environment, Dor Intratável, Psicossomática, Ambulatório Hospitalar, Estresse Psicológico, Meio Social

## Abstract

A distinguishing characteristic of the biomedical model is its compartmentalized view of man. This way of seeing human beings has its origin in Greek thought; it was stated by Descartes and to this day it still considers humans as beings composed of distinct entities combined into a certain form. Because of this observation, one began to believe that the focus of a health treatment could be exclusively on the affected area of the body, without the need to pay attention to patient’s subjectivity. By seeing pain as a merely sensory response, this model was not capable of encompassing chronic pain, since the latter is a complex process that can occur independently of tissue damage. As of the second half of the twentieth century, when it became impossible to deny the relationship between psyche and soma, the current understanding of chronic pain emerges: that of chronic pain as an individual experience, the result of a sum of physical, psychological, and social factors that, for this reason, cannot be approached separately from the individual who expresses pain. This understanding has allowed a significant improvement in perspective, emphasizing the characteristic of pain as an individual experience. However, the understanding of chronic pain as a sum of factors corresponds to the current way of seeing the process of falling ill, for its conception holds a Cartesian duality and the positivist premise of a single reality. For phenomenology, on the other hand, the individual in his/her unity is more than a simple sum of parts. Phenomenology sees a human being as an intending entity, in which body, mind, and the world are intertwined and constitute each other mutually, thus establishing the human being’s integral functioning. Therefore, a real understanding of the chronic pain process would only be possible from a phenomenological point of view at the experience lived by the individual who expresses and communicates pain.

## Abstract (pt)

**Resumo:**

Uma característica marcante do modelo biomédico é a visão de homem compartimentalizada na qual se embasa. Esta forma de ver o ser humano teve origem no pensamento grego, foi afirmada por Descartes e permanece até hoje como sendo este ser composto por entidades distintas que se combinam em determinada sorte. Como fruto desta constatação passou-se a acreditar que o foco da atenção de um tratamento de saúde poderia ser dado especificamente à área do corpo acometida, sem que fosse necessária atenção à subjetividade do doente. Compreendendo a dor como mera resposta sensorial, este modelo não pôde alcançar o que seria a dor crônica, por esta se tratar de um processo complexo podendo existir independente de lesão tecidual. A partir da segunda metade do século XX, quando se torna impossível negar a interferência entre psique e soma, surge a compreensão de dor crônica que se tem hoje: de uma experiência individual, resultado de uma soma de fatores físicos, psicológicos e sociais, não podendo por isso ser abordada de modo desvinculado ao indivíduo que a expressa. Este entendimento permitiu um grande avanço de ponto de vista por ressaltar sua característica de vivência particular. Entretanto, a compreensão de dor crônica enquanto soma de fatores faz jus ao modo atual de compreensão do adoecimento mantendo em sua concepção a dualidade cartesiana e a premissa positivista de se voltar a uma única realidade. Para a fenomenologia, em contra partida, o indivíduo em sua unidade é mais que a mera soma de partes. Entende o ser humano como *entidade intenciona*l, onde corpo, mente e mundo são entrelaçados e constituem-se mutuamente, estabelecendo, assim, um tipo de funcionamento completamente integral do ser humano. Deste modo, o real entendimento de um processo de dor crônica só seria possível a partir de um olhar fenomenológico da experiência como vivida pelo indivíduo que a expressa e comunica.

## Introduction

This paper is the result of a theoretical collaboration carried out by professionals from the field of psychology in its interface with health, who base their interventions on the phenomenological model.

This is a critique of the way chronic pain processes have been understood by health sciences and through phenomenological thinking it draws attention to the proposal of articulating and strengthening the phenomenological perspective with health sciences.

Chronic pain is a complex object that cannot be understood based on the biomedical paradigm, because it is an individual experience and involves aspects other than the physical ones [1]. Today, pain is seen as the sum of physical, psychological, and social factors and, for this reason, it cannot be approached separately from the individual who expresses it.

The comprehension of chronic pain as a sum of factors corresponds to the current way of seeing the process of falling ill, which reflects the western view of man. This compartmentalized way of seeing the individual has its origin in Greek thought, was stated by Descartes, and remains to this day viewing the human being as composed of distinct entities combined into a certain form. As a result of this observation, one began to believe that the focus of a health treatment could be exclusively on the affected area of the body, without the need to pay attention to the individual’s identity–who and how the patient could be–, a feature of the biomedical model of health.

However, not even models founded on holistic alternatives succeeded in their aim to understand what the experience of this type of pain could be. The expansion of this understanding into the psychological and social factors of the ways of falling ill represented by psychoanalysis–and later by psychosomatics–and the proposition of a different health model–the biopsychosocial–holds a Cartesian duality and the positivist premise of pursuing a single reality based on natural science methods [2].

This can be observed especially in the definition of pain in use today, proposed by IASP (International Association for the Study of Pain): “an unpleasant sensory and emotional experience associated with actual or potential tissue damage, or described in terms of such damage” [3]. However, in spite of the mind-body split still present in its concept, this understanding has allowed a significant improvement in its perspective, for emphasizing that pain is a private, individual experience. Pain cannot be expressed through diagnosis technology, it is what it means for the subject that experiences it.

In the experience of living with pain, the individual’s world is affected and the experiences that constitute this individual also define the origin and expression of his/her pain. The indivisible world of a patient includes his/her pain, but from the point of view of health it continues to be compartmentalized.

At heart, phenomenology tries to retrieve precisely the understanding of this individual who, in his/her unity, is more than a simple sum of parts. The individual seen as an intending entity would not be a mere product of the influences of the world, but rather a part of it, in which body, mind, and the world would be intertwined and constitute each other mutually, thus establishing the human being’s integral functioning.

Therefore, to get a fuller understanding of a chronic pain process a phenomenological outlook is hard to be avoided.

The path outlined in this introduction is based on the connection between the evolution of knowledge about pain and the development of medicine, in an attempt to discuss the efforts of objectifying and subordinating pain to science in the history of both pain and science, from supernatural interferences to the focus materialized on the body. Concurrently, there is a critique of how models to understand health–products of this evolution–were not capable of achieving a real understanding of chronic pain. The next section introduces the first records about pain as an expression of something that transcends the physical body, analyzing the influence of the divided man of Cartesian thought and the attempts to retrieve a subjectivity that has been underestimated for centuries. These attempts failed, because they continued to be objectified and distant from the individual. Following, we introduce phenomenological thought as an effort to retrieve a view of man closer to reality and as the possibility of understanding what suffering from chronic pain would be. In this topic, we also discuss how distant the phenomenological perspective is from both theory and practice with regard to health.

## About chronic pain

Pain is probably one of the oldest and most universal forms of stress and one of the earliest sufferings of humankind. Understanding it is one of the major concerns of humanity; however, despite the efforts and the fact that it is as old as humanity itself, pain neither has been completely understood nor can be totally controlled [4,5].

International compendiums and the most different scientific articles about pain are unanimous about the difficulty to define it and emphasize how hard it is to treat chronic pain [6-10].

Considered as an integral part of life, pain has always been present throughout the development of the human being, exerting a protective function on the body. Associated with diseases, inflammatory processes, accidents, and medical or surgical procedures, pain functions as a warning sign indicating that something is not well. This adaptive aspect is of great value for survival, because it is based on the sensation of pain that individuals are motivated to move away from fire and pointed objects or to seek treatment for certain symptoms. Individuals with a genetic disorder called *congenital insensitivity to pain*, although able to distinguish tactile sensations such as temperature and pressure, do not have pain among their experiences and, for this reason, are more prone to accidents and may not reach adulthood [11].

Some systems of pain classification have been developed with the didactic aim of categorizing pain and creating a terminology to facilitate communication between researchers, healthcare teams, and patients. The most common classification used today considers pain according to its duration and includes acute and chronic pain [12-14].

Acute pain has a relatively short duration, from some minutes to some weeks. It derives from tissue damage, inflammatory processes, or illnesses [7,15]. It is felt at some moment in life by the majority of the individuals and has a positive meaning, because it indicates injury or illness. Some examples are post-operative pains, pains associated with medical procedures in general, pains derived from scratches or extensive trauma, some headaches, acute myocardial infarction, labor, and many other clinical conditions. The experience of this type of pain is a complex process that activates a series of neurophysiological, hormonal, and psychological mechanisms, characterizing an alarm reaction and preparing the body for the fight and flight response [4].

However, there is a type of pain that, even having been associated with disease or injury, persists after treatment [7,16]. More specifically, according to the American Pain Society and the International Association for the Study of Pain, it is the pain that persists beyond the usual time for tissue cicatrization, for a period of more than three months [17,18]. This type of pain is no longer seen as a symptom, it is considered an illness in itself, called chronic pain [7]. Much more comprehensive than a persistent symptom, it is a complex physiopathological, diagnostic, and therapeutic situation. Individuals who suffer from chronic pain hardly show any improvement, regardless of the therapeutic resources used to treat it, putting in check physicians’ knowledge and patience [16]. It is typical of chronic pain patients to submit to a series of treatments, and even to unnecessary surgeries, and their pilgrimages to several doctor offices is universal [19-21].

In general, the constant presence and the long duration of pain are deeply disturbing. Pain becomes the focus of the individual’s attention and makes a large part of his/her activities more difficult. Frequently, the individual ends up with altered mobility, sleep, sexual life, and humor, and can also show low self-esteem, negative thoughts, a hopeless perception of life, and changes in his/her family, work, and leisure relationships [22]. Gooberman-Hill and colleagues [23] state that pain that lasts more than three months is considered disabling, affecting several levels of the subject’s activity, as well as his/her social interactions and, consequently, his/her well-being.

Craig [15] states that pain can be highly destructive of the psychological and social well-being of a patient, who can become seriously debilitated and under severe stress caused by failures in self-healing biological mechanisms and unsuccessful attempts of self-control and medical treatments. The longer pain lasts, the higher the individual’s probability to be depressed, aloof, irritated, and more and more worried and persistent in his search for relief.

Rheumatoid arthritis, postherpetic neuralgia, degenerative spine conditions, osteoarthritis, AIDS, migraine, diabetic neuropathy, and phantom pain are examples of chronic pain action [16,20,24] as well as cancer, in which, for some authors, pain refers to a specific category: progressive chronic pain [25-27]. The chronification of pain may derive from a progressive chronic disease and emerge only at a certain moment of disease development, or derive from postoperative symptoms or sports injuries.

Chronic pains can also be classified as *organic* or *emotional*, according to the presence or absence of current or previous tissue damage. Organic pains can be *nociceptive*, when there is a peripheral painful stimulus originating from viscera or tissues, or *neuropathic*, when resulting from damage at any level of the central or peripheral nervous system. In the so-called emotional pains, the existence of neuropathic or nociceptive stimuli is not acknowledged [28,29].

In the economic sphere, it is estimated that the cost to treat patients with chronic pains exceeds the total cost to treat patients with heart disease, cancer, and AIDS [30]. Prolonged pain is among the major causes of absence from work, sick leaves, disability retirement, severance pay, and low productivity. It is a problem of public health for its prevalence, high cost, and negative impact on the quality of life of patients and their families [31].

## Comprehensions of pain and the theoretical models of health

### From supernatural to submission to science

According to Rey [32], the beginning of medicine is related to pain relief, and there would be no medical art without its search for effective remedies or its effort to interpret pain with the purpose of naming the organ affected or predicting the cure for the disease. Therefore, it was in contemporary medicine that chronic pain became a medical object derived from a historical and epistemological construction [1]. This development encourages reflection on the origin of this process and on how the biomedical reasoning itself is structured.

At its emergence, medical science was closely linked to witchcraft, from which it separates when it becomes able to explain for itself the causes of diseases and to propose their cure [33,34].

In many cultures, before the beginning of the Middle Ages, pain was perceived as a result of the influence of entities that were outside the body. Physical ailments in general were seen in Mesopotamia as sin or impurity. Disease was punishment inflicted by gods and could claim either the sinner or his/her whole family [35] . In Ancient Egypt, pain was thought to be caused by the spirits of the dead residing in the body of the individual affected by pain. In Classical Greece, with Hippocrates, and in Ancient Rome, with Galen, the first steps were taken to explain pain rationally [36]. However, the Greek tragedy of the fifth century B.C. valued a brutal and concrete interpretation of pain, contributing in a certain way to provide an irreplaceable testimony of particular expressions [32].

The Middle Ages were marked by the extended hegemony of Galenism, as a perfectly coherent system that covered all problems of medicine, and by the importance of the Arabic medicine of Avicenna. At that time, pain begins to play an important role in the prognosis of diseases, besides indicating the site affected [32].

The end of the Dark Age is marked by the use of a new class of chemical agents to control pain and the eventual recognition of the power of opium [36]. However, the changes in conceptions from Antiquity were small. It was necessary to wait for the construction of another model of science, with different demands, to see changes in the foundations of knowledge [32].

At the onset of the eighteenth century, the growth of medical and scientific knowledge was levered by the development of the microscope and by the use of dissection techniques in autopsies, which contributed to the understanding of the human body functioning. It was also in this period that it was discovered that certain diseases were caused by microorganisms, which fostered the development of antiseptic and anesthetic techniques and the progress of surgery [37].

Thus, the new science that takes shape in the Classical Age tends to abandon occult forces, beliefs and purely nominal explanations permanently: *“In this context (…) medicine itself tried to envisage the human body as a complex machine which could be compared to an ensemble of ropes, levers, and pulleys. It tried to reason in a ‘geometric fashion’, i.e. by rigorously stringing together all its propositions and accepting only that which could be proven”*[32] (p. 99).

It is in this period that there is an improvement in the substances used to control pain. The use of chloroform, nitrous oxide, and ether as anesthetic in surgeries, of cocaine as a regional anesthetic [38], and the identification of neuronal receptors and nervous impulses transmission eventually make pain to be treated as an exclusively biological phenomenon, to be explained physiologically [39].

Deriving from these advancements in the medical field, and based on the belief that mind and body function separately, a model of conceptualizing health and disease emerges, the so-called biomedical model. This model proposes that all diseases or physical disorders can be explained by disturbances in physiological processes, which, on their turn, can be explained by biochemical imbalance, viral or bacterial infections [37,40,41]. Disease would be an exclusively bodily ailment, independent of psychological and social processes. This conception was broadly accepted during the nineteenth and twentieth centuries and represents to this date the prevailing view in the medical field [42].

### The divided man: the biomedical model

In much the same way as in the historical development of medicine, knowledge related to pain was deepened and approaches physical suffering separately from emotional and social events [35,37].

The view that mind and body function separately is reinforced as knowledge improvements bring evidence that the causes of diseases and the possibilities of cure are in the body itself. The body deprived of its identity would suffice and became the object of medicine. In this context arises the biomedical model.

However, the idea of separation between body and mind is not simply a consequence of this scientific evolution process. Since the first writings about health and disease, which date back to 500–300 B.C., mind and body are seen as separate and unrelated entities [42]. Hippocrates and his students devoted efforts to eradicate the vestiges of the magical-religious way of thinking about the human body. As a challenge to healers, Hippocrates taught physicians (*iatros*) to treat only what was observable or palpable [43].

Although this tradition has its origin in Greek thought, it was the philosopher and mathematician René Descartes (1596–1650) who formulated more clearly the ideas that immediately precede the contemporary biomedical concepts about the human body [43] and that became a landmark of modern rationality [44]. For Descartes, man was composed of two substances: the soul (abstract, spiritualistic, thinking, and indivisible) and the body (the physical part, concrete, divisible). Soul was different from body in such a way that it could exist independently. The only interaction between both was through the pineal gland, regarded as the seat of the soul [45]. Likewise, since religious matters were mainly related to the issues of human soul, from that moment on it was clear that science and religion would also be set apart [46].

According to Leder [47], Descartes had a profound interest in immortality and thought the main objective of his studies was the development of a new medicine, one that could overcome diseases, postponing the arrival of death. For years, the investigation methodology chosen by Descartes was the dissection of animal bodies, with the objective of understanding their functioning, since he believed that body’s life was modeled on the workings of an inanimate machine [47]. Consequently, this human body deprived of life eventually became a mark of Cartesian metaphysical and scientific explorations.

In this way Descartes, a devout Catholic, was able to preserve the soul as the domain of theology and to legitimate the body as the domain of science. The so-called Cartesian dualism thus freed biology to pursue a radically materialist thinking, but withdrew the mind (soul) from clinical practice for the next 300 years [43].

In an attempt to show that modern medicine is deeply rooted in Cartesian thought, Leder [47] reviews how the development of medicine maintained its focus on an inanimate body, the corpse: “Medical education still begins with the dissection of a cadaver, just as the clinical case ends in the pathologist’s lab” (p. 121). With medical technology, diagnostic instruments such the use of stethoscope, blood tests, and image tests allow an access to the living body similar to that achieved only by corpse dissection.

Leder points out that patients are frequently treated as corpses during physical examinations, when they are asked to assume the pose of a dead body: flat, passive, mute, and naked; if they are called upon to express themselves, it is always in search for their mechanical functioning. The knee is tapped to provoke reflexes, the abdomen is poked to see if pain ensues, and the patient is asked to breathe deeply to hear whether the lungs produce audible sounds. As in the Cartesian conclusion that the living body can be treated as a machine, medicine proposes forms of treatment that are mechanical as well: to a cardiac patient, a drug that will bring physiological changes; exercises may be recommended to strengthen the heart muscle; and, in case surgery is necessary, the body will be opened up and some vessels will be replaced. The physician uses means to alter body functioning, as one would do with a mechanical thing, replacing parts and regulating processes [47].

In this context are established perceptions of pain that Turk [14] and colleagues classify as belonging to the Restrictive Theories, among which the Specificity Theory stands out [48]. According to this theory, a specialized transmission system carries messages from exclusive skin receptors for pain to a pain center in the brain. Pain is regarded as a specific sensation, with its own sensory equipment, independent of other senses, which would result in a direct relation, with no possibility of variation between the physical stimulus and the sensation perceived by the individual; therefore, pain intensity would be proportional to the extension of tissue damage [7,49,50].

This proposition for the mechanism of pain as a stimulus–response reaction was initially made by René Descartes himself, in 1644, and has reached the refinement described above through improvements made by Muller and Von Frey in the following centuries [51]. This theory had significant gaps, since it was not able to explain pains not necessarily associated with injury or those that persisted after having been treated–such as phantom pain and peripheral neuralgia–and it did not admit the action of components other than the mechanical ones in the process. However, this same theory was responsible for the emergence of several surgical methods to manipulate chronic pain by nerve sectioning, which are still in use to block pain sensation [5].

### The twentieth century and the attempts to connect the human machine parts: the biopsychosocial model

From the beginning of the twentieth century and with the emergence of new schools of thought, Cartesian dualism and science itself began to be questioned. It was in that century that an epistemological break occurs in the history of science, when the reductionist, mechanicist, and determinist view of the world of Newtonian physics and Cartesian thought, prevailing since the seventeenth century, is refuted.

Moreover, because of treatment improvement, disease pattern has also changed. With the advancement of medicine, the main health problems, previously associated with infectious diseases, began to be related to chronic diseases. External ailments, which would have earlier caused the individual’s death, became treatable and curable. Thus, it was observed that treatment success is increasingly dependent on patient’s behaviour [37].

Therefore, the interference of emotional issues is gradually admitted at the origin of the process of falling ill. This interference is seen as the exteriorization of an internal conflict with the external world [52] or as the influence of emotional and social aspects on treatment adherence and evolution. These conceptions were not considered by the biomedical model to understand health and disease processes.

Freud’s revolutionary ideas, gathered under the name of *psychoanalysis*, call attention to and also mark this epistemological break [39]. Freud’s work on conversion hysteria with patients who showed symptoms of physical diseases without organic cause attracts the curiosity of physicians and researchers to the study of the interaction of emotional and bodily processes [37]. It also contributes to understand the circumstances that culminate in the concretization of suffering in a psychological or somatic manifestation based on the investigation of the conflict origin [53]. However, Freud has never written about psychogenesis [54]. Well before him, in 1818, Heinroth coined the term *psychosomatics*, which soon fall into disuse. It was retrieved only a century later, as *psychosomatic medicine* by Felix Deutsch [55]. The main concern of this new branch of medicine was to find the symbolism of some diseases and elements to understand the relation between emotional states and organic symptoms.

Consequently, psychosomatics has been involved with the comprehension of the relation between social and psychological factors, biological and physiological functions, as well as with the development of several physical diseases, encouraging research development and emphasizing psychoanalytical interpretations about specific health problems. For such, psychosomatics was based on Franz Alexander’s idea about basic conflicts typical of diseases such as ulcers, migraines, asthma, hypertension, cancer, and others, and sometimes on the concept of psychological profiles coined by Flanders Dunbar, who considered that there were specific personal profiles predisposed to specific diseases [37,56].

However, in spite of considering the interaction of psychological and social processes in disease states, emphasizing the individual’s history, psychosomatics is now a model susceptible to be questioned. Its concept carries a heavy semantic load rooted in the philosophical dualism of Cartesian thought–as the view of man that is being discussed here–, which have spread through modern rationality to the concept of disease as a whole. To think of psychosomatics is to assert–as in its own definition, the connection of psyche and soma–the existence of two separate entities, disregarding the notion of man as a unity; it is to treat pathologies as processes of organic or psychological order, and not as results of the interaction of an integral body, in which mind and body are one, which makes this concept redundant [44,57].

Therefore, psychosomatics, as Turato [58] states, carries an idea of monodirectionality, a dichotomic emphasis, and induces to an impervious individualization. It thus fails to encompass an understanding of the person, to seek his/her totality and his/her form of expression, because it remains committed to search explanations based on causes or factors and on stimulus and response [56].

The twentieth century also marks the evolution of the concept of pain. Because of the gaps in the Specificity Theory, Melzack and Wall’s Theory of the Gate Control, published in 1965 [48], has gained credibility for its comprehensiveness. This theory holds that pain is regulated by a “gate” that can be opened or closed by impulses from peripheral nerves or from the central nervous system, increasing or decreasing perceived pain. This mechanism of gates would be influenced by a series of factors, such as mood states and environmental stimuli, which would induce the gates of the bands of spinal cord nerve fibers to open, so that pain impulses could reach the brain, or to close, with the aim of blocking them. The physiological description of this mechanism would not be appropriate here, because there is no scientific evidence of its functioning and because it is probably incorrect; however, this theory has revolutionized knowledge about pain, for eliciting other elements of the process, such as the individual’s emotional state and the integration of new therapeutic approaches, rather than being explained simply as an afferent sensory experience [59].

Based on formulations such as this one, pain is now considered by the International Association for the Study of Pain (IASP) as “an unpleasant sensory and emotional experience associated with actual or potential tissue damage, or described in terms of such damage” [3]. This definition admits the existence of the individual’s subjectivity and, consequently, of his/her particular way of expression. As described by Lima and Trad [1], pain is in the body, in the mind, in life-history, in the everyday, in the lifeworld, *i.e.*, it is multidimensional. According to Harding and colleagues [60], chronic pain is a complex experience influenced by the individual’s sociocultural environment, by his/her beliefs, expectations, attitudes, and the meaning he/she assigns to his/her own pain, as well as by biological factors.

It is in this same context, in which body, mind and social environment are partners in the process of falling ill, that the biopsychosocial model emerges. This model is understood as a humanistic approach that studies behaviors and experiences, aiming at uncovering underlying meanings and interpretations, and that acknowledges the uniqueness and complexity of the human experience [2]. For this reason, it demands a multidimensional evaluation of the individual, taking into account the aspects of the disease itself; the patient’s behavior; the social, cultural, and family context in which he/she lives; and the health system itself [61].

However, we perceive that, as in psychosomatics, in spite of valuing the individual’s emotional aspects and social context, this model continues to reproduce a model of thought based on Cartesian dualism. For Traverso-Yépez [2], the biopsychosocial model shares with the biomedical model the positivist premise of pursuing a single reality to be uncovered by the methods of natural science, which denotes a merely palliative stance in relation to the biomedical perspective. The biopsychosocial model adds psychological and social dimensions to the understanding of the process of falling ill more as rhetoric than as a legitimate practice [62,63].

Consequently, so far, it seems there is no theoretical model for the processes of falling ill–especially for the experience of chronic pain–which includes the object of which it speaks: the individual. Therefore, a new way of thinking and understanding the human being is needed.

## Phenomenology and the intentionality of the body that hurts

### The person in pain

It is based on the concept of *intentionality*, which Brentano [64] has retrieved from the Scholasticism and was later enunciated by thinkers such as Husserl and Merleau-Ponty, that we propose a way of seeing the individual opposed to the Cartesian model.

Husserl’s appropriation of the concept of intentionality describes this principle as consciousness that is invariably consciousness of something, and that consciousness of something is consciousness only when directed to an object. In the same way, the object can only be defined in its relation with consciousness, it is always object-for-a-subject. An object only exists and has meaning for a certain consciousness [65]. Intentionality is each and every relation between the individual and his world; it is not in the individual nor in a certain object, but in the relation between both.

Likewise, Merleau-Ponty’s philosophy proposes that, for understanding the relationship of man with the world, we must transcend dichotomies. Merleau-Ponty approaches a model of man that is not inserted in the western dualist thought either, but speaks of a subject constituted by the world, which constitutes the world, and that one does not exist exclusively for the other, but is part of the other. Man’s boundaries with the world are “blurred”, denying the dichotomy subject-object [66,67].

For Merleau-Ponty, intentionality is the meaning that emerges from the contact of the individual with the world and the others in a dialectical relationship. It is a type of primordial–not rational, carnal, or bodily–perception. The intending consciousness establishes a type of integral functioning of the individual, in which body, mind, and the world are intertwined and constitute themselves mutually.

The singularity of Merleau-Ponty’s understanding of intentionality is in showing that the relationship subject-world is essentially mediated by the body: “I am conscious of the world through my body” [68] (p. 122). This philosopher introduces a new way of understanding the body, which he calls *lived body*, because it is through this body that man opens himself to the world and, therefore, perceives this world and himself [68].

Thus, we understand that the *lived body* is an intending entity [47]. From the perspective that each and every relationship of the individual with his/her world is intentional and that all contact of the individual with his/her world is mediated by his/her body, we conclude that an individual is always a human being in this living body, which is permanently in relation with the world around him/her and that it is only based on this interaction that he/she constitutes his/her own world, with his/her own meanings and perspectives.

Drew Leder [47] states that the body is a material entity constituted of organs that function in a specific way, but that it also plays a subjective role. Retaking the term used by Merleau-Ponty, Leder reaffirms that the lived body is an intertwining between perception and what is perceived, it is intentional and material. When he opposes to the Cartesian paradigm, and once again echoing Merleau-Ponty, Leder affirms that the body is not simply an object in the world, but an intending entity in which the world emerges. If the body is alive, it is related, and the individual is made of these relations.

The way of seeing man described here is clearly opposed to the model deprived of intentionality and subjectivity proposed by Descartes. Understanding pain exclusively from an anatomopathological perspective, as something situated in a certain part of the body, is to not understand what feeling pain is in human terms. The body that hurts is an individual constituted by the world that surrounds him/her, influences him/her, and is influenced by him/her at every moment, and it has unique characteristics–which, without attention, may limit therapeutic intervention.

Viewed in this light, pain, when it occurs, calls the individual’s attention to the affected part of his/her body and may control him/her as no other bodily experience. According to Leder [69], because of pain the individual’s time and space experiences change. Perception turns to the sensation of the moment, with a focus on the site of pain. Moreover, pain can limit locomotion and eliminate interest in other stimuli. Whereas a healthy individual is able to explore the past through memories and fantasize the future, for a person who suffers from chronic pain, his/her past of pain is all that he/she wants to forget, and a future without pain is impossible to conceive [69].

As a process of falling ill, chronic pain can thus be understood as a way of being in the world, and it is not necessarily located in a certain site [70]. However, it affects the individual as a whole, for seizing his/her attention, raising questions, causing suffering, changing his/her role in the family, changing his/her work situation, limiting his/her possibilities of leisure, and so on.

The perspective brought about by phenomenology seems essential to understanding the point of view we want to achieve today to comprehend the ways of falling ill and the constitution of chronic pain–its origin and presence–in the reality of individuals.

It is not possible to reduce an individual’s experience of pain to a number on a scale (as usually requested in some patient evaluations), just as one does not overcome a state of chronic pain solely by interventions based on experience or on the most recent scientific discoveries. It is not possible to provide quality help to a person whose history is unknown. To understand pain, it is necessary to give voice to the patient’s experience [71].

Thus, we can conclude that chronic pain does not exist in isolation: there are individuals who manifest chronic pain, and to understand it, we need to observe it from this individual’s perspective. In addition, we cannot think of a single form of process manifestation, since each individual is unique and his/her ways of understanding, signifying, and expressing are singular as well.

In Madjar’s words [71]:

To understand pain we need to understand the person in pain and a phenomenological gaze can help us to do that. The key is our attentiveness to the lived experience of the person in pain, and our willingness, individually and as members of health care teams, to work as much *with* as *on* our patients. The cognitive and technical work of pain diagnosis and treatment needs to go hand in hand with the supportive, and the affirming acts that make possible for the patient’s voice to be heard and to be valued (pg. 275).

### A perspective to be affirmed

The phenomenological perspective associated with healthcare, although addressed by authors such as Baron [70], Leder [69], Toombs [72] and Svenaeus [73], among others, is still rare in scientific publications.

In a survey (carried out on April 24, 2013) of the electronic database PubMed to write this paper, the association of the terms *phenomenological* and *pain* retrieved 129 studies, which are listed as material published in important medical sciences journals in the past five years. However, among these papers, just five include discussions from a phenomenological perspective; most (112) are related to research carried out from traditional health perspectives and use qualitative phenomenological methodology. The remaining twelve articles did not match our search terms: they concerned the general use of the term *phenomenological* or were related to psychological pain. Consequently, we conclude that phenomenology is present and is rather common in the field of health as a research methodology, but not as a way of theoretical articulation, of new possibilities of seeing the human being.

Within the scope of healthcare interventions and contact with patients, the phenomenological understanding of the individual is also away from professionals’ practice. In these settings, we observe that the biologicist etiology, the fragmented conception of health, and the imperative and ruling character of a positivist view of science continue to be favored, overlooking the relevance of social, psychological, and ecological aspects as mediators in health-disease processes [2]. And even more distant is the understanding of these factors as intertwined. Lima and Trad [74], in an attempt to comprehend the senses and meanings assigned to chronic pain by the physicians of the pain management service of two large university hospitals in Brazil, point to a still unidimensional view of the constitution of chronic pain.

Although acknowledging the importance of psychological intervention in the process, it is only when medical understanding cannot pinpoint and explain pain that the psychologist is mentioned in the process. It is only when known physiological mechanisms are not detected that attention is given to the psychological and social determinants of pain [1]. There is a gap between health professionals’ point of view and the experience lived by the patient. Professionals try to separate pain from anxiety, depression, suffering, and from other emotional reactions, to the point of assigning these aspects of the human experience to different expertise [71]. Patients, on the other hand, do not experience pain as a pure sensation; for them, pain “arrives as a complete package… painful, miserable, disturbing…” [75] (p. 149).

### Final remarks

In its attempt to understand, explain, and intervene in the ways of falling ill–particularly of chronic pain–, the reductionist view of the current models of health is not able to grasp the complexity of such phenomena. With the advancement of medical sciences and the observation of physical determinants in some illnesses, in addition to the parallel cultural strengthening of Cartesian thought and positivist science, we observe an excision of all other influence on the body beside the physical, visible ones.

In face of recent evidence, based on the emergence of new theories and clinical observation, health professionals begin to understand how the determinant for adherence to and efficacy of modern treatments proposed depends more on the individual’s psychosocial context than on medical technology. Then, they begin to conceive a new form of thinking about their patients, considering that, beyond patients’ physical complaint, there is something subjective that interferes in the origin, expression, and management of their illnesses.

However, in the daily routine of health services, they still think of a divided individual, composed of the sum of biological, psychological, and social parts, to the detriment of a unified view. In practice, this individual is treated by different professionals in specialized walk-in services and ends up suffering from interventions that still favor his/her physical complaints or overvalue certain aspects of his/her subjectivity.

Based on what was proposed, we understand that the phenomenological thought enables a way of thinking that goes beyond what has already been theoretically structured. Reaffirming the individual’s functioning as integral, unthinkable or unmanageable in separate instances, this perspective sees a human being constituted of the world in which he/she lives and of his/her experiences, who expresses in the experience of chronic pain who he/she is.

As the understanding of chronic pain is a challenge to science in its tireless attempts to objectify pain, we understand that this type of pain can be easily described in phenomenological terms, since the only way to understand the other’s pain is through his/her communication of his/her subjective experience. According to Scarry [76] (p. 13), “to have pain is to have *certainty*; to hear about pain is to have *doubt*”. It is in the phenomenological gaze that lies the possibility of understanding experience as it was lived by the individual who communicates it [71].

However, this philosophical point of view is not directly related to the theories and interventions of health psychology disciplines; it falls to healthcare professionals and researchers to reflect to articulate what the theory may affirm and what it can do for their practice, besides how to combine such theory with an already present theoretical restlessness to overcome psychosomatic theories and the biopsychosocial model.

Therefore, our intention is to provoke a critical look at the theories correlated to models of understanding health, as well as at professional interventions, services, and stances, in addition to providing suggestions of new theoretical and intervention models. We raise here the possibility of using the foundation proposed by phenomenology to structure a new model, a new way of thinking about health.

## Competing interests

The authors declare that they have no competing interests.

## Authors’ contributions

DDL is responsible for the conception, protraction and drawing up of the article. VLPA is responsible for the drawing up and critical revision of the article. ERT is responsible for the critical revision of the article. All authors read and approved the final manuscript.

## Authors’ information

DDL

Psychologist, psychotherapist in the Person Centered Approach. Masters in Medical Sciences and PhD candidate in Medical Sciences by the Department of Medical Psychology and Psychiatry, State University of Campinas. Member of the Laboratory of Clinical–Qualitative Research (Unicamp/CNPq).

VLPA

Psychologist, psychotherapist in the Person Centered Approach, PhD in Education by the State University of Campinas. Researcher at the Post Doctoral Program at the Medical Sciences Faculty – UNICAMP. Member of the Laboratory of Clinical–Qualitative Research (Unicamp/CNPq).

ERT

Professor of the Department of Medical Psychology and Psychiatry, State University of Campinas. Coordinator of the Laboratory of Clinical–Qualitative Research (Unicamp/CNPq).
